# Pediatric severe asthma with fungal sensitization is mediated by steroid-resistant IL-33

**DOI:** 10.1016/j.jaci.2015.01.016

**Published:** 2015-08

**Authors:** Susana Castanhinha, Rebekah Sherburn, Simone Walker, Atul Gupta, Cara J. Bossley, James Buckley, Nicola Ullmann, Ruth Grychtol, Gaynor Campbell, Marco Maglione, Sergio Koo, Louise Fleming, Lisa Gregory, Robert J. Snelgrove, Andrew Bush, Clare M. Lloyd, Sejal Saglani

**Affiliations:** a Department of Respiratory Paediatrics, Royal Brompton Hospital, London, United Kingdom; b Leukocyte Biology, NHLI, Imperial College London, London, United Kingdom; d Airways Disease, NHLI, Imperial College London, London, United Kingdom; c Department of Respiratory Paediatrics, Kings College Hospital, London, United Kingdom

**Keywords:** Severe asthma, fungal sensitization, pediatric, IL-33, innate immunity, steroid resistance, ABPA, Allergic bronchopulmonary aspergillosis, AHR, Airway hyperresponsiveness, BAL, Bronchoalveolar lavage, HDM, House dust mite, ICOS, Inducible costimulator, ILC, Innate lymphoid cell, MMP-9, Matrix metalloproteinase 9, SAFS, Severe asthma with fungal sensitization, sIgE, Specific IgE, SPT, Skin prick test, STRA, Severe therapy-resistant asthma

## Abstract

**Background:**

The mechanism underlying severe asthma with fungal sensitization (SAFS) is unknown. IL-33 is important in fungus-induced asthma exacerbations, but its role in fungal sensitization is unexplored.

**Objective:**

We sought to determine whether fungal sensitization in children with severe therapy-resistant asthma is mediated by IL-33.

**Methods:**

Eighty-two children (median age, 11.7 years; 63% male) with severe therapy-resistant asthma were included. SAFS (n = 38) was defined as specific IgE or skin prick test response positivity to *Aspergillus fumigatus*, *Alternaria alternata*, or *Cladosporium herbarum*. Clinical features and airway immunopathology were assessed. Chronic exposure to house dust mite and *A alternata* were compared in a neonatal mouse model.

**Results:**

Children with SAFS had earlier symptom onset (0.5 vs 1.5 years, *P* = .006), higher total IgE levels (637 vs 177 IU/mL, *P* = .002), and nonfungal inhalant allergen-specific IgE. Significantly more children with SAFS were prescribed maintenance oral steroids (42% vs 14%, *P* = .02). SAFS was associated with higher airway IL-33 levels. In neonatal mice *A alternata* exposure induced higher serum IgE levels, pulmonary IL-33 levels, and IL-13^+^ innate lymphoid cell (ILC) and T_H_2 cell numbers but similar airway hyperresponsiveness (AHR) compared with those after house dust mite exposure. Lung IL-33 levels, IL-13^+^ ILC numbers, T_H_2 cell numbers, IL-13 levels, and AHR remained increased with inhaled budesonide during *A alternata* exposure, but all features were significantly reduced in ST2^−/−^ mice lacking a functional receptor for IL-33.

**Conclusion:**

Pediatric SAFS was associated with more oral steroid therapy and higher IL-33 levels. *A alternata* exposure resulted in increased IL-33–mediated ILC2 numbers, T_H_2 cell numbers, and steroid-resistant AHR. IL-33 might be a novel therapeutic target for SAFS.

Atopy is a prominent characteristic of severe pediatric asthma,^1^ and many allergens can exacerbate disease, including fungal exposure both outside and indoors.^2,3^ In adults fungal sensitization is associated with increased asthma severity, morbidity, and mortality, including higher rates of hospital and intensive care unit admission.^2^ In children with persistent symptoms, asthma with fungal sensitization was associated with worse disease severity, increased bronchial reactivity, increased airway eosinophilic inflammation,^4^ and more exacerbations.^5^

Although an association between fungal sensitization and increased morbidity is apparent, whether such an association is causal remains unconfirmed.^6,7^ Recently, a subphenotype of severe asthma with fungal sensitization (SAFS) has been described in adults^8,9^ and is associated with reduced lung function and increased morbidity.^10,11^ The definition of SAFS includes severe asthma treatment at British Thoracic Society level 4 or equivalent; fungal sensitization demonstrated by a positive skin prick test (SPT) response or specific IgE (sIgE) to at least one of 7 fungi (ie, *Aspergillus fumigatus*, *Alternaria alternata*, *Cladosporium herbarum*, *Penicillium chrysogenum*, *Candida albicans*, *Trichophyton mentagrophytes*, or *Botrytis cinerea*); and no evidence of allergic bronchopulmonary aspergillosis (ABPA^8^; a very rare diagnosis in asthmatic children). Little is known regarding the pathogenesis of pediatric SAFS. A single case report described dramatic improvement with oral itraconazole.^12^ A retrospective review of adults with ABPA or SAFS in whom therapy with itraconazole had failed showed a significant improvement in asthma severity when treated with the second-line antifungal agents voriconazole or posaconazole.^13^ A randomized controlled trial of itraconazole in adults with SAFS showed a significant improvement in quality of life.^14^ However, a further trial of voriconazole in adults with moderate-to-severe asthma and sensitization to *A fumigatus* did not show any benefit.^15^ Recently, IL-33 has been shown to contribute to the development of fungal exacerbation of allergic airways disease in an adult murine model after chronic house dust mite (HDM) exposure.^16^ However, mechanisms underlying chronic fungal exposure and sensitization remain unknown. We hypothesized that fungal sensitization in children with severe therapy-resistant asthma (STRA) is associated with more severe disease and is mediated by the innate cytokine IL-33. We investigated the clinical and pathologic features of fungal sensitization in children with STRA and delineated mechanistic differences underlying chronic fungal and HDM exposure in a neonatal mouse model of allergic airways disease.

## Methods

### Subjects

Children aged 6 to 16 years with STRA were recruited from the Royal Brompton Hospital (London, United Kingdom). They had already undergone a detailed assessment to optimize adherence and address underlying modifiable factors as much as possible.^17^ STRA was defined as previously described,^1^ as persistent chronic symptoms, exacerbations, or both despite high-dose inhaled corticosteroids (beclomethasone equivalent ≥800 μg/d), long-acting β-agonists, and either current or a previous failed trial of leukotriene receptor antagonists. Two groups were defined: (1) patients with SAFS with sIgE or positive SPT responses to any of *A fumigatus*, *A alternata*, or *C herbarum* and (2) non–fungus-sensitized patients (non-SAFS) with negative sIgE levels and SPT responses to all these fungal allergens. Sensitivity to other fungi is not routinely tested in our department. The study was approved by the local research ethics committee, and informed parental consent and child assent were obtained.

### Clinical assessment

Age at onset of symptoms, medications, and symptom scores (Asthma Control Test)^18^ were recorded. Spirometry with bronchodilator reversibility was performed according to American Thoracic Society/European Respiratory Society guidelines.^19^

### Atopy

Atopy was assessed based on total serum IgE levels, sIgE levels, and SPT responses to *A fumigatus*, *A alternata*, *C herbarum*, HDM, cat, dog, grass pollen, peanut, milk, and egg. Atopy was defined as at least 1 positive sIgE RAST result (≥0.35 kUI/L) or SPT response (wheal diameter, ≥3 mm) to aeroallergens (HDM, cat, dog, and grass pollen) and quantified as the sum of these aeroallergens (sIgE levels and SPT-induced wheal diameters) and the sum of all (nonfungal) allergens (aeroallergens and peanut, milk, and egg).^20^

### Airway inflammation and remodeling

Fraction of exhaled nitric oxide at 50 mL/s, (values <24 ppb were considered normal) and results of induced sputum cytology^1^ were used to assess eosinophilic airway inflammation noninvasively. Bronchoalveolar lavage (BAL) and endobronchial biopsy inflammation and IL-33 expression and remodeling (reticular basement membrane thickness and airway smooth muscle mass) were also quantified, as previously described.^1,21^

### Assessment of BAL mediators from pediatric samples

The T_H_2 cytokines IL-4, IL-5, and IL-13 and other proinflammatory cytokines (IL-6 and IL-8) were measured in BAL fluid by using a multiplex cytokine assay (Luminex, Bio-Rad, Hemel Hempstead, United Kingdom). IL-33 was not available as part of the assay, and reliable data could not be obtained by using commercially available ELISAs. Therefore the molecular size and quantity of IL-33 was determined by means of Western blotting. BAL fluid protein levels were determined by using the BCA assay (Pierce, Cheshire, United Kingdom). Five hundred nanograms of total protein of each sample was loaded and run on a reducing gel before transfer to a polyvinylidene fluoride membrane and blocking in 5% nonfat milk TBS-Tween. Primary antibody (IL-33; R&D Systems, Abingdon, United Kingdom) was incubated overnight at 4°C at 1:1000 dilution. Secondary antibodies (New England Biolabs, Hitchin, United Kingdom) were used at 1:2000 dilution and incubated for 1 hour at room temperature. Bands were developed by with ECL-Plus (Pierce) and imaged on film (Kodak, Rochester, NY). Densitometric analysis was performed with ImageJ software (http://imagej.nih.gov/ij/). Matrix metalloproteinase 9 (MMP-9) activity was determined by using Novex 10% zymogram gelatin gels (Invitrogen, Carlsbad, Calif).

### Neonatal allergic airways disease

Neonatal BALB/c or ST2^−/−^ mice underwent intermittent intranasal HDM (20 μg for the first 2 weeks, followed by 25 μg) or saline exposure from day 3 of life for 3 weeks, as previously described.^22^ *A alternata* was administered intranasally 3 times per week (5 μg for the first 2 weeks, followed by 10 μg in the third week). Airway hyperresponsiveness (AHR) to methacholine was determined by using the forced oscillation technique in anaesthetized and tracheostomized mice 4 hours after final challenge with HDM or 18 hours after the final *A alternata* challenge, as previously described.^22^ In experiments to assess the effects of steroid therapy, mice were treated with 0.6 mg/kg intranasal budesonide (Pulmicort Respules; AstraZeneca, London, United Kingdom) or PBS (10 μL) daily during the period of allergen exposure. All experiments were performed in accordance with UK Home Office guidelines.

### Tissue processing and analysis

Serum, BAL fluid, and lung tissue were collected and analyzed, as previously described.^22^ Paired antibodies for murine IgE, (BD Biosciences, Oxford, United Kingdom), IL-13, IL-33 (R&D Systems), IL-4, and IL-5 (PharMingen, Oxford, United Kingdom), were used in standardized sandwich ELISAs, according to the manufacturer's protocol. Serum HDM- and *A alternata*–specific IgE was analyzed, as previously described.^22^

### Flow cytometry

Disaggregated lung cells were restimulated with 500 ng/mL ionomycin and 50 ng/mL phorbol 12-myristate 13-acetate in the presence of brefeldin (BD PharMingen, Oxford, United Kingdom). Cells were stained for CD3, CD4, IL-13 (eBioscience, San Diego, Calif), or lineage-negative cocktail (anti-CD3 [17A2], anti-CD45R [B220], [RA3-6B2], anti-CD11b [M1/70], anti–TER-119 [TER-119)], anti–Ly-G6 [Gr-1, RB6-8C5; eBioscience], CD45 [eBioscience], and inducible costimulator [ICOS; BioLegend, London, United Kingdom]). Labeled cells were acquired on a BD Fortessa (BD Biosciences) and analyzed by using FlowJo software (TreeStar, Ashland, Ore).

### Statistical analysis

There are no data to inform a power calculation for the pediatric data, and therefore the sample size was opportunistic. Statistical analysis was performed with GraphPad Prism software (version 5; GraphPad Software, La Jolla, Calif), and all data were analyzed by using nonparametric tests. Between-group differences were analyzed with the Mann-Whitney test. If more than 2 groups were present, the Kruskal-Wallis test, followed by a Dunn correction was used; χ^2^ or Fisher exact tests were used, where appropriate (categorical data); and correlations were assessed by using the Spearman correlation. Values are expressed as medians with ranges.

## Results

### Clinical features of SAFS in children

Eighty-two patients (median age, 11.7 years; range, 4-17 years) were included. Thirty-eight (46%) of 82 had SAFS. Demographic data and clinical differences between the 2 groups are summarized in Table I. Children with SAFS were predominantly boys and had significantly earlier age of symptom onset. Patients with SAFS had significantly higher total serum IgE levels and sum of inhalant nonfungal allergen SPT responses and sIgE results (Table I). Current symptoms and lung function, including bronchodilator reversibility, were similar in children with and without fungal sensitization. Airway inflammation and remodeling were similar in children, regardless of fungal sensitization (see Table E1 in this article's Online Repository at www.jacionline.org). Although lung function and pathologic parameters were comparable in both groups, significantly more children with SAFS were prescribed maintenance oral steroids to achieve similar clinical status (*P* = .02, Table I).

Because SAFS was associated with higher serum IgE levels, the clinical response to anti-IgE antibody therapy was assessed. Twenty-eight of the 82 children included underwent a clinically indicated trial of omalizumab, and 10 of 28 had SAFS. Eight (80%) of 10 children with SAFS and 11 (64%) of 18 without SAFS had a clinically beneficial response to omalizumab (not significant, Table I).

### Children with SAFS had significantly more pulmonary IL-33 and MMP-9 activity compared to non-SAFS

**Fig E1:**
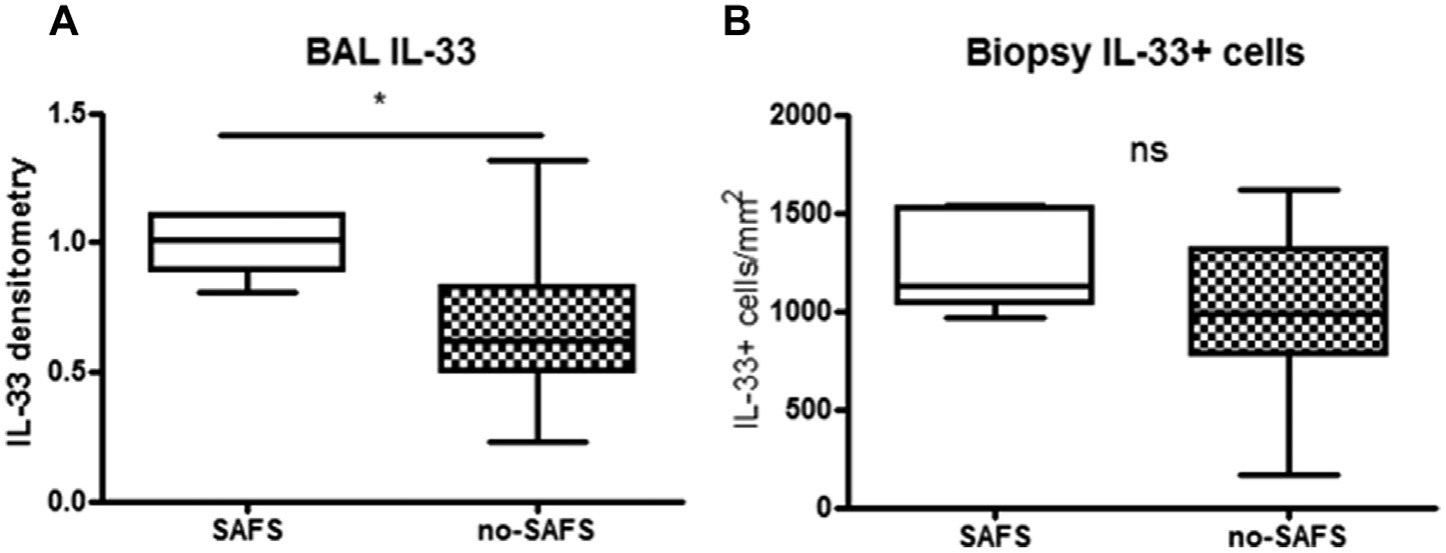
IL-33 levels were quantified in BAL fluid by means of Western blotting **(A)**, and IL-33^+^ cells stained by means of immunohistochemistry were quantified in the submucosa of endobronchial biopsy specimens (**B**; n = 14 children). Group differences were assessed by using the Mann-Whitney *U* test: *P < .05. *ns*, Not significant.

**Fig E2:**
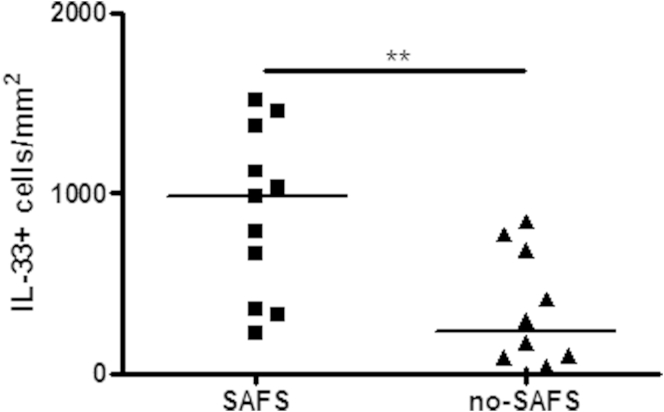
Endobronchial biopsy specimens from children aged 6 to 16 years with severe asthma receiving high-dose inhaled steroids only were stained by using immunohistochemistry for IL-33 expression. IL-33^+^ cells in the submucosa were counted and expressed per area of submucosa in children with SAFS compared with children with severe asthma without fungal sensitization (*no-SAFS*; n = 2). Group differences were assessed by using the Mann-Whitney *U* test: **P < .01.

**Fig 1:**
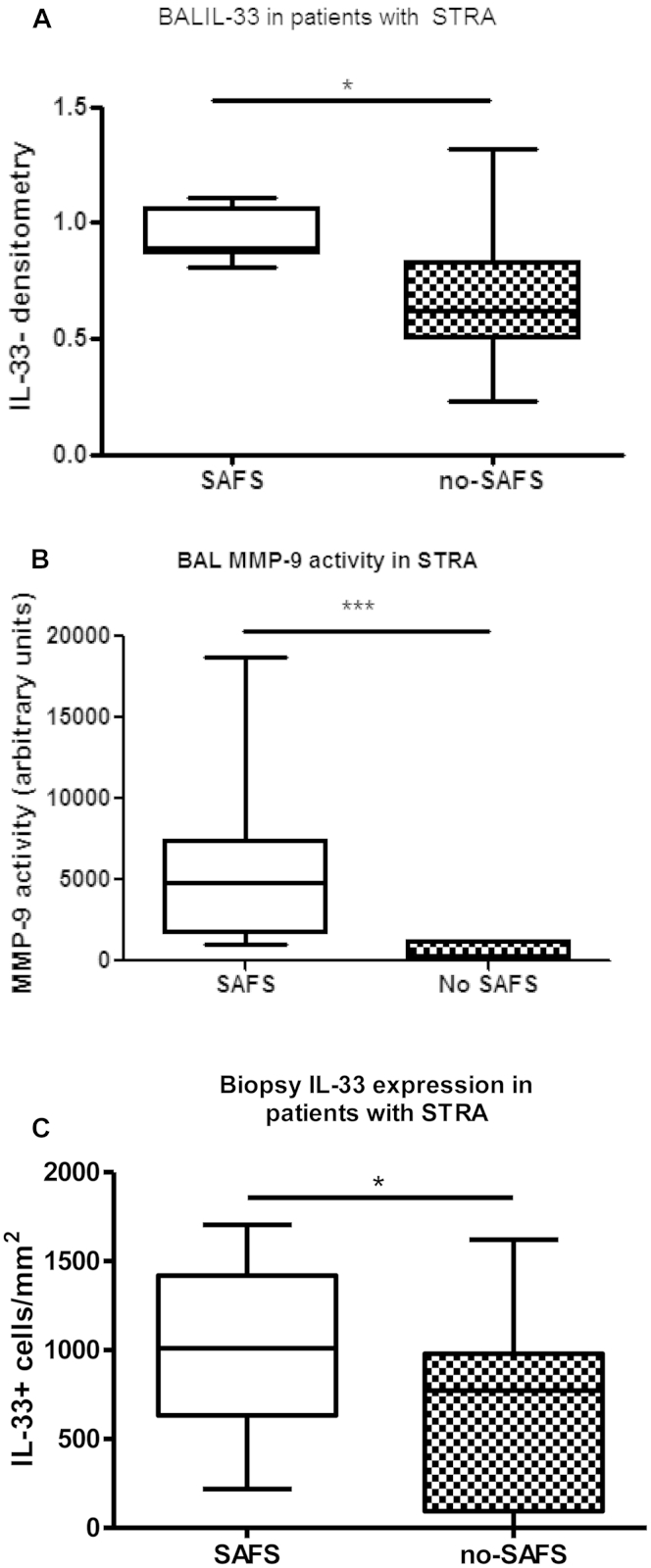
Increased IL-33 levels **(A)** and MMP-9 activity **(B)** in BAL fluid and increased submucosal IL-33^+^ cell numbers in endobronchial biopsy specimens **(C)** from children with STRA and SAFS compared with those without fungal sensitization *(no SAFS)*. There were 18 specimens for BAL data, and 33 specimens for biopsy data. *P < .05 and ***P < .001.

We have previously shown *A alternata*–induced exacerbations of allergic airways disease are mediated by IL-33^16^ and that IL-33 is relatively steroid resistant.^21^ Because more children with SAFS were receiving maintenance oral steroids, we quantified IL-33 levels in BAL fluid and endobronchial biopsy specimens comparing children with and without fungal sensitization. Children with SAFS had significantly greater BAL fluid and tissue IL-33 levels compared with those who did not have evidence of fungal sensitization (Fig 1, *A* and *C*, and see Fig E1 in this article's Online Repository at www.jacionline.org). This remained true when children receiving maintenance oral steroids were excluded (see Fig E2 in this article's Online Repository at www.jacionline.org), suggesting IL-33 is induced by fungal sensitization. A recent adult murine fungal exacerbation model demonstrated a relationship between IL-33 levels and the protease and proremodeling mediator MMP-9.^16^ In concurrence, BAL fluid MMP-9 activity assessed by using gelatin zymography was significantly higher in children with SAFS (Fig 1, *C*).

### Comparison of chronic *A alternata* exposure to HDM exposure in neonatal mice

**Fig 2:**
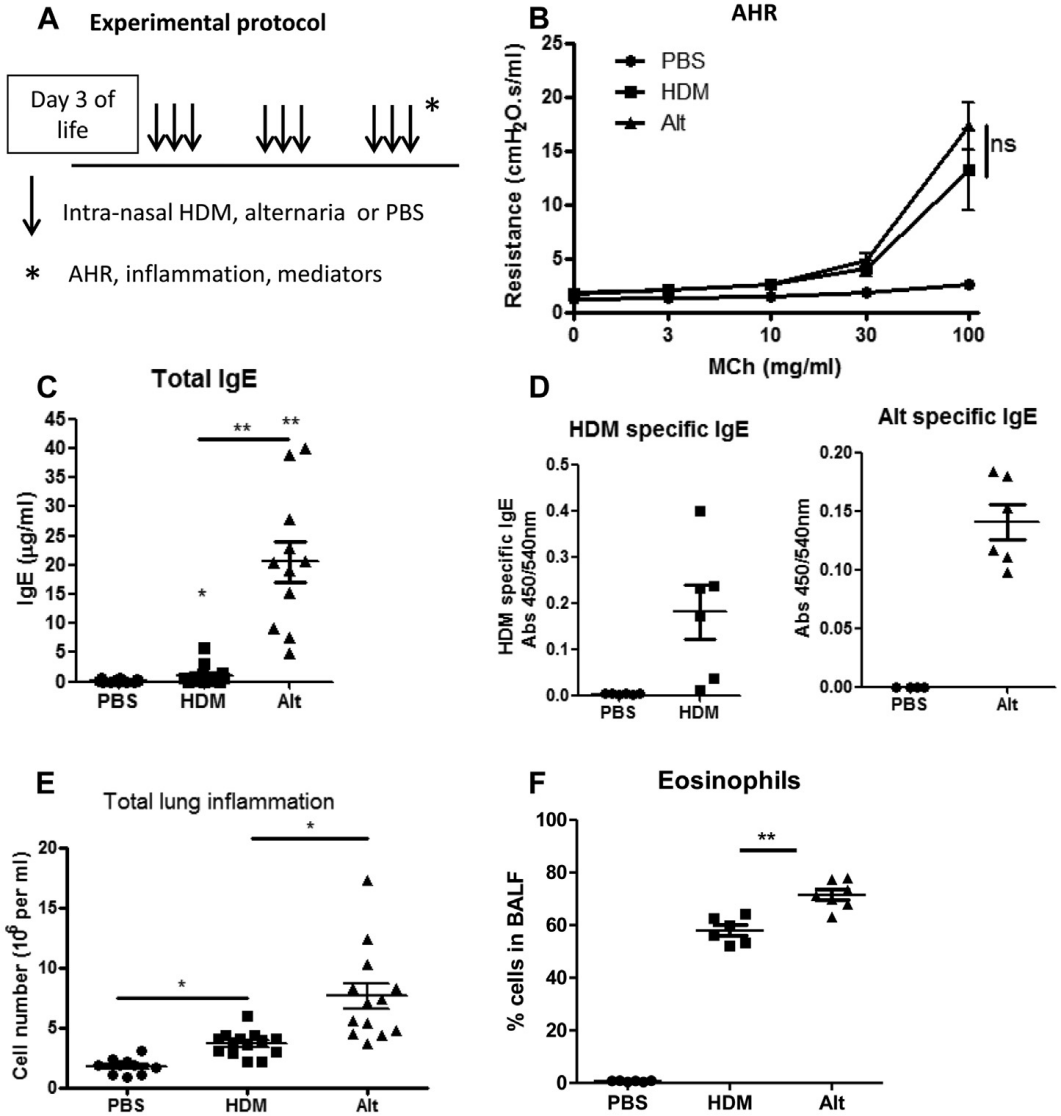
Fungal exposure in neonatal mice resulted in more severe atopy and inflammation than HDM exposure, but AHR was similar with both allergens. Neonatal BALB/c mice were challenged with intranasal HDM (20 μg for the first 2 weeks and then 25 μg) or *A alternata* (*Alt*; 5 μg for the first 2 weeks and then 10 μg) for 3 weeks **(A)**, and assessments of AHR **(B)**, total serum IgE levels **(C)**, allergen-specific IgE levels **(D)**, total pulmonary inflammation **(E)**, and eosinophilic inflammation **(F)** were made. Data are representative of 2 experiments (n = 4-8 per group). *P < .05 and **P < .01. *ns*, Not significant.

Because most children with STRA are polysensitized to several aeroallergens, it is difficult to disentangle mechanisms attributable to fungal sensitization alone. *A alternata* exposure was compared with HDM exposure in neonatal mice (Fig 2, *A*) to determine specific features of allergic airways disease resulting from fungal sensitization. Both allergens induced similar AHR (Fig 2, *B*). However, total serum IgE levels (Fig 2, *C*), allergen-specific IgE levels (Fig 2, *D*), and total pulmonary (Fig 2, *E*) and eosinophilic (Fig 2, *F*) inflammation were significantly higher after *A alternata* exposure compared with that seen after HDM exposure.

### Chronic *A alternata* exposure resulted in increased IL-33 levels compared to HDM exposure

**Fig E3:**
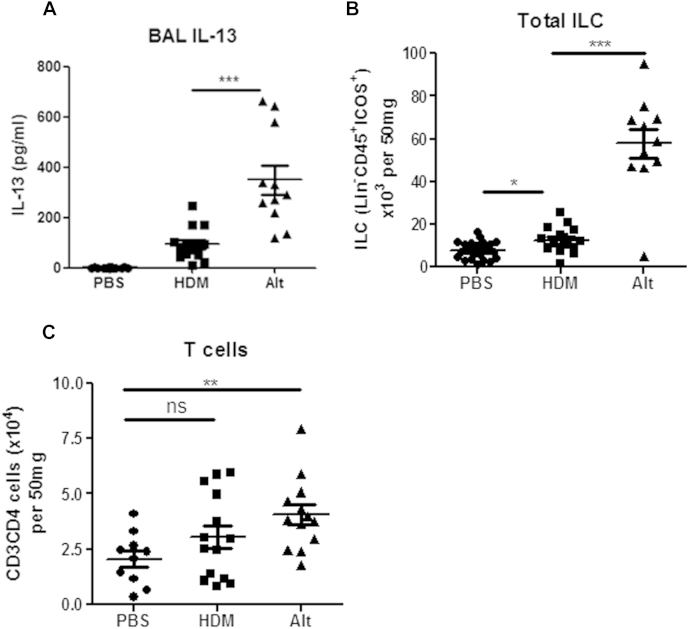
Neonatal BALB/c mice were exposed to intermittent intranasal *A alternata (Alt)*, HDM, or saline (PBS) for 3 weeks. Total BAL fluid IL-13 levels were measured by means of ELISA **(A)**, and numbers of Lin^−^CD45^+^ICOS^+^ ILCs **(B)** and CD3^+^CD4^+^ T cells **(C)** were quantified by means of flow cytometry. *P < .05, **P < .01, and ***P < .001. *ns*, Not significant.

**Fig 3:**
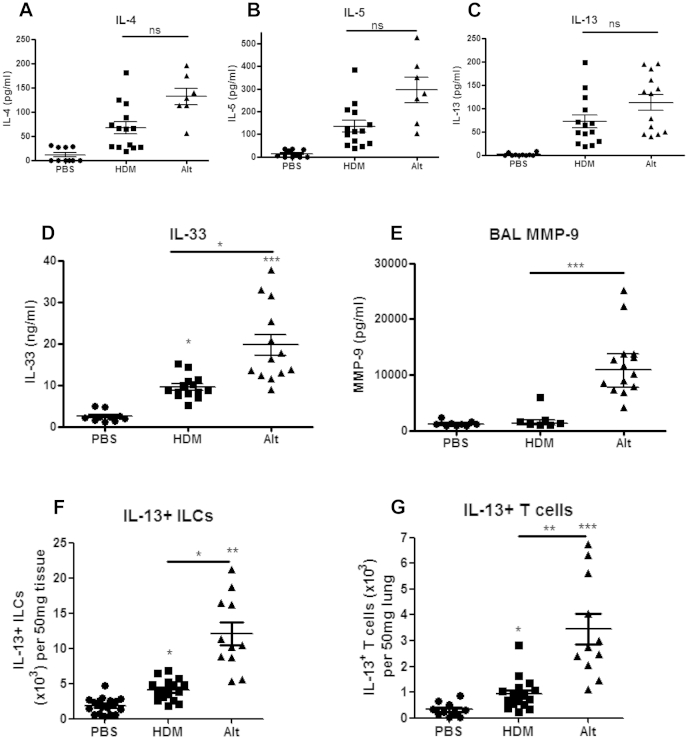
*A alternata* exposure resulted in significantly increased lung IL-33 levels, but lung IL-13 levels were similar to those after HDM exposure. Pulmonary IL-4 **(A)**, IL-5 **(B)**, IL-13 **(C)**, and IL-33 **(D)** levels; BAL fluid MMP-9 levels **(E)**; IL-13^+^ Lin^−^CD45^+^ICOS^+^ ILC numbers **(F)**; and IL-13^+^CD3^+^CD4^+^ T-cell numbers **(G)** in neonatal BALB/c mice exposed to intranasal saline (PBS), HDM, or *A alternata (Alt)* for 3 weeks are shown. Data are representative of 2 experiments (n = 4-8 per group). *P < .05, **P < .01, and ***P < .001.

Because chronic *A alternata* exposure was associated with enhanced IgE levels and inflammation, we determined T_H_2 cytokine levels after fungal exposure. Pulmonary IL-4, IL-5, and IL-13 levels were similar after *A alternata* and HDM exposure (Fig 3, *A-C*). However, BAL fluid IL-13 levels were significantly higher after *A alternata* exposure (see Fig E3, *A*, in this article's Online Repository at www.jacionline.org). Pulmonary IL-33 levels were significantly higher in mice exposed to *A alternata* compared with levels in those exposed to HDM (Fig 3, *D*), and BAL fluid MMP-9 levels were also significantly higher after *A alternata* exposure than after HDM exposure (Fig 3, *E*).

Because IL-33 is known to induce type 2 ILCs in the context of adult models of allergic airways disease^23^ and levels of IL-33 were significantly higher after *A alternata* compared with HDM, we investigated whether type 2 ILCs might represent the cellular source of IL-13 after *A alternata* exposure. Interestingly, both pulmonary Lin^−^CD45^+^ICOS^+^ ILCs and CD3^+^CD4^+^ T cells were significantly increased after *A alternata* exposure, and numbers were significantly higher when compared with those after HDM exposure (see Fig E3, *B* and *C*). Specifically, numbers of IL-13^+^ ILCs and IL-13^+^ T_H_2 cells were significantly higher in mice exposed to *A alternata* compared with those seen in mice exposed to HDM (Fig 3, *F* and *G*). These data suggest fungal exposure results in enhanced adaptive and innate type 2 immune responses in neonatal mice.

### Neonatal ST2^−/−^ mice exposed to *A alternata* had significantly lower AHR and serum IgE levels

**Fig E4:**
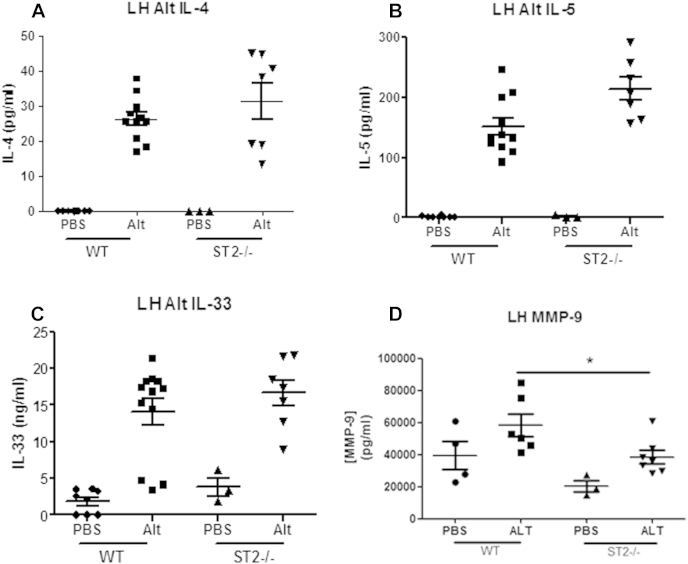
Neonatal ST2^−/−^ and wild-type mice were exposed to intermittent intranasal *A alternata* (*Alt*; 5 μg for the first 2 weeks and then 10 μg) or saline (PBS) from day 3 of life for 3 weeks. Levels of IL-4 **(A)**, IL-5 **(B)**, IL-33 **(C)**, and MMP-9 **(D)** were measured in lung homogenates *(LH)* by means of ELISA. Data are representative of 2 experiments (n = 6-8 per group). *P < .05.

**Fig 4:**
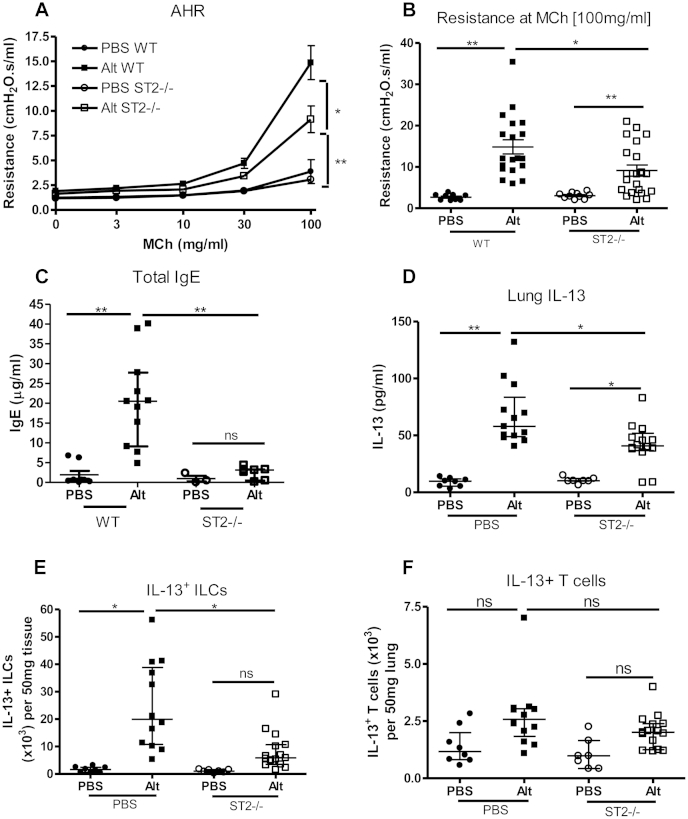
Neonatal ST2^−/−^ mice have significantly reduced allergic airways disease after *A alternata* exposure compared with wild-type mice. Neonatal mice were exposed to intermittent intranasal *A alternata* (*Alt*; 5 μg for the first 2 weeks and then 10 μg) or saline (PBS) from day 3 of life for 3 weeks. AHR (**A** and **B**), total serum IgE levels **(C)**, pulmonary IL-13 levels **(D)**, and IL-13^+^Lin^−^CD45^+^ICOS^+^ ILCs **(E)** and IL-13^+^CD3^+^CD4^+^ T cells **(F)** were assessed. Data are representative of 2 experiments (n = 6-8 per group). *P < .05 and **P < .01. *MCh*, Methacholine; *ns*, not significant.

Neonatal ST2^−/−^ mice, which lack a functional receptor for IL-33, were exposed to *A alternata* for 3 weeks to confirm whether IL-33 was a key pathogenic mediator in the development of SAFS. ST2^−/−^ mice had significantly reduced AHR in response to *A alternata* (Fig 4, *A* and *B*), lower serum IgE levels (Fig 4, *C*), lower pulmonary IL-13 levels (Fig 4, *D*), and fewer IL-13^+^ ILCs (Fig 4, *E*) compared with those found in wild-type mice. Interestingly, there were no changes in lung IL-13^+^ T-cell numbers (Fig 4, *F*) and IL-4, IL-5, or IL-33 levels (see Fig E4, *A-C*, in this article's Online Repository at www.jacionline.org) in ST2^−/−^ mice, but MMP-9 levels were significantly reduced (see Fig E4, *D*). These data suggest that AHR induced by chronic fungal exposure is mediated by IL-33 through induction of IL-13^+^ ILCs.

### AHR is unaffected by steroid therapy after *A alternata* exposure but is completely abrogated after steroids and HDM exposure

**Fig E5:**
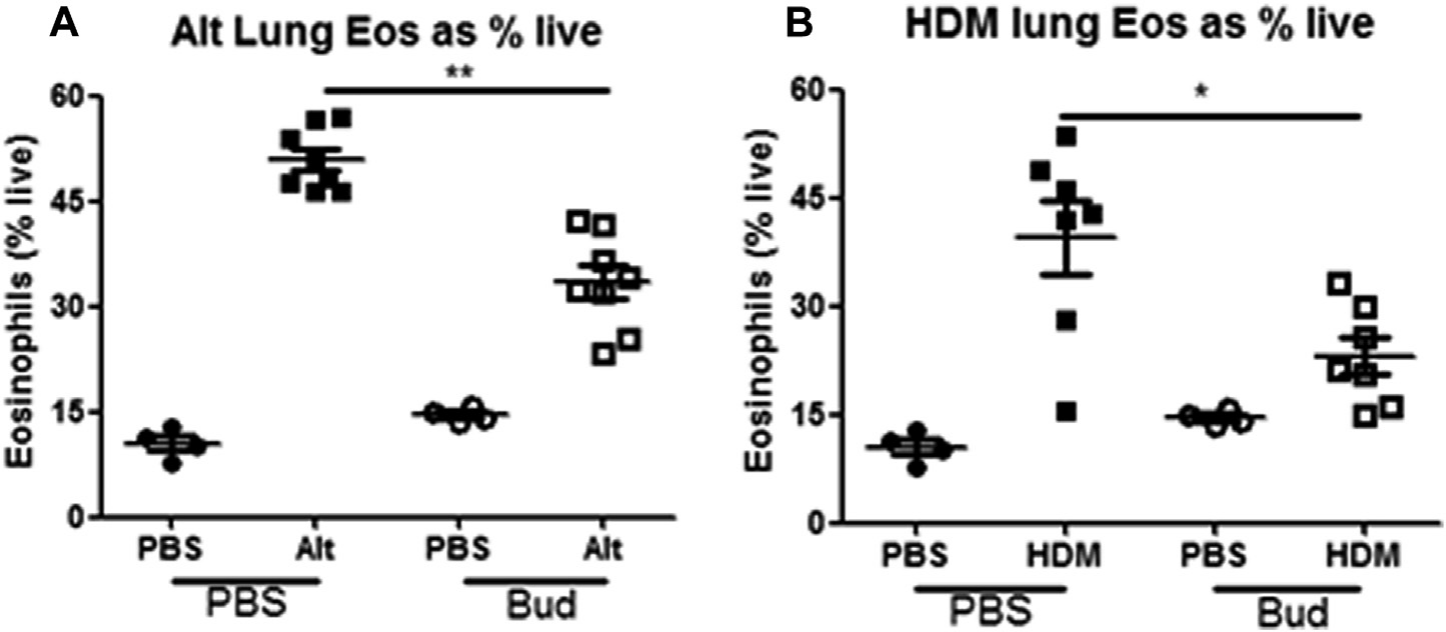
Neonatal BALB/c mice were exposed to intranasal saline (PBS), HDM, or *A alternata (Alt)* from day 3 of life for 3 weeks, with concomitant intranasal budesonide (0.6 mg/kg) or PBS from day 10 of life for 2 weeks (prevention regimen). These data relate to the same experiment shown in Fig 5. Numbers of eosinophils were quantified by means of flow cytometry in lung digests after *A alternata* exposure **(A)** and HDM exposure **(B)** with and without budesonide (*Bud*; n = 4-6 per group for PBS and n = 6-8 per group for HDM or *A alternata*. *P < .05 and **P < .01.

**Fig 5:**
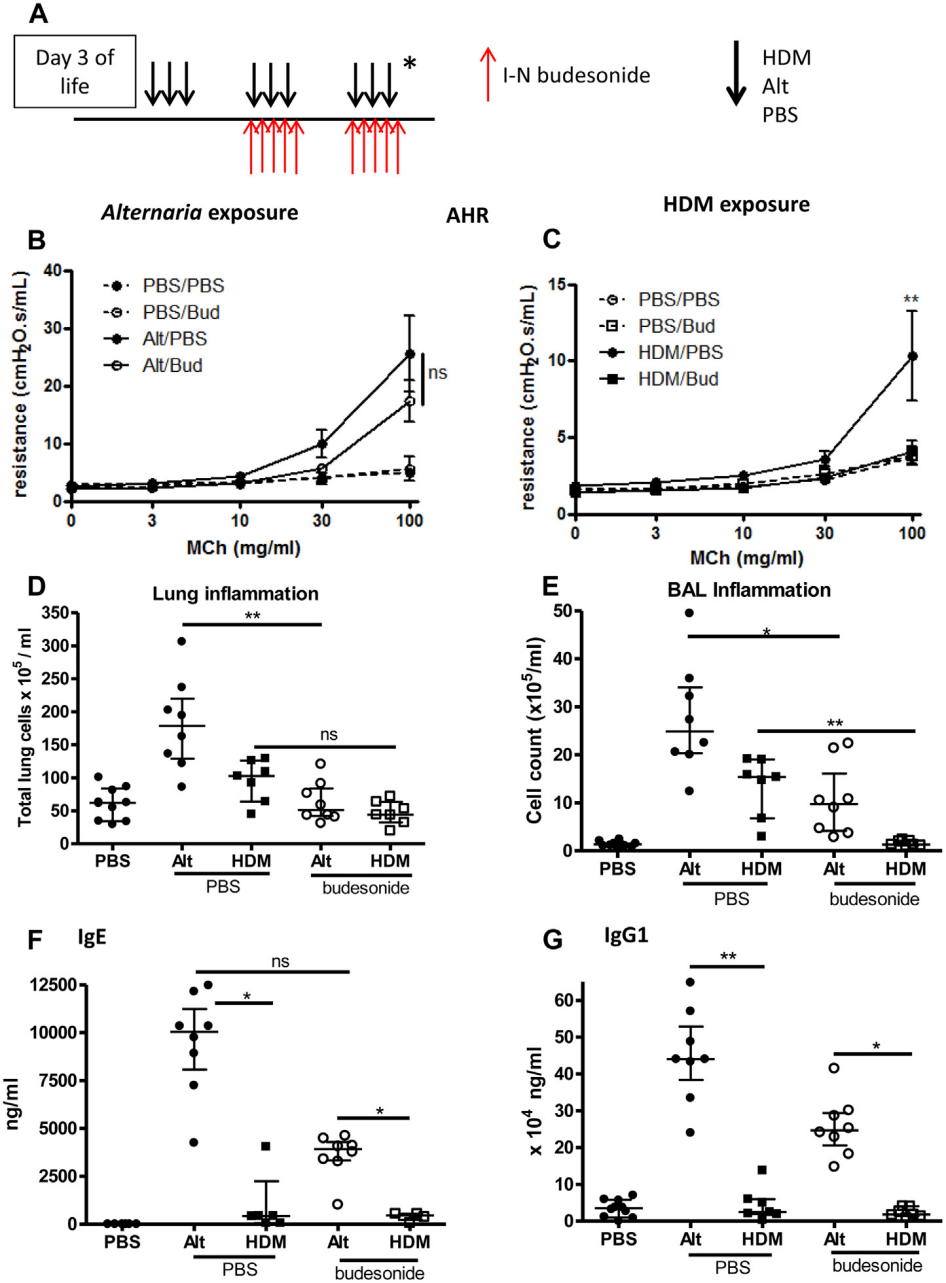
AHR is unaffected by steroids after *A alternata* exposure, whereas inflammation and serum IgE levels are reduced. **A,** Neonatal BALB/c mice were exposed to intranasal *(I-N)* saline (PBS), HDM, or *A alternata (Alt)* from day 3 of life for 3 weeks, with concomitant intranasal budesonide (0.6 mg/kg) or PBS from day 10 of life for 2 weeks (prevention regimen). **B-G,** AHR (Fig 5, B and *C*), total lung inflammation (Fig 5, *D*), BAL inflammation (Fig 5, *E*), and serum immunoglobulin levels (Fig 5, *F* and *G*) were assessed (n = 4-6 per group [PBS] or n = 6-8 per group [HDM or Alt]. *P < .05 and **P < .01. *MCh*, Methacholine; *ns*, not significant.

We have shown that IL-33 is more steroid resistant than IL-13.^21^ Because *A alternata* exposure resulted in higher IL-33 levels in the murine model and significantly more children with SAFS were prescribed oral steroids to achieve similar symptom control and lung function (Table I), we investigated whether *A alternata* exposure induces a steroid-resistant phenotype of allergic airways disease. Concomitant intranasal administration of budesonide and allergen to neonatal mice (Fig 5, *A*) had no effect on AHR after *A alternata* exposure (Fig 5, *B*), but AHR was completely abrogated after HDM exposure (Fig 5, *C*). *A alternata*– and HDM-induced total lung inflammation were reduced to control levels after steroid treatment (Fig 5, *D*), whereas eosinophilic inflammation was partially reduced (see Fig E5 in this article's Online Repository at www.jacionline.org). BAL inflammation was only partially reduced after steroids and *A alternata* exposure but completely abrogated after HDM exposure and steroids (Fig 5, *E*). Despite steroid therapy, total serum IgE and IgG_1_ levels remained significantly increased after *A alternata* exposure, whereas both were reduced to control levels after HDM exposure (Fig 5, *F* and *G*).

### ILC2s, T_H_2 cells, and IL-13 levels remain elevated after *A alternata* exposure despite steroid therapy

**Fig 6:**
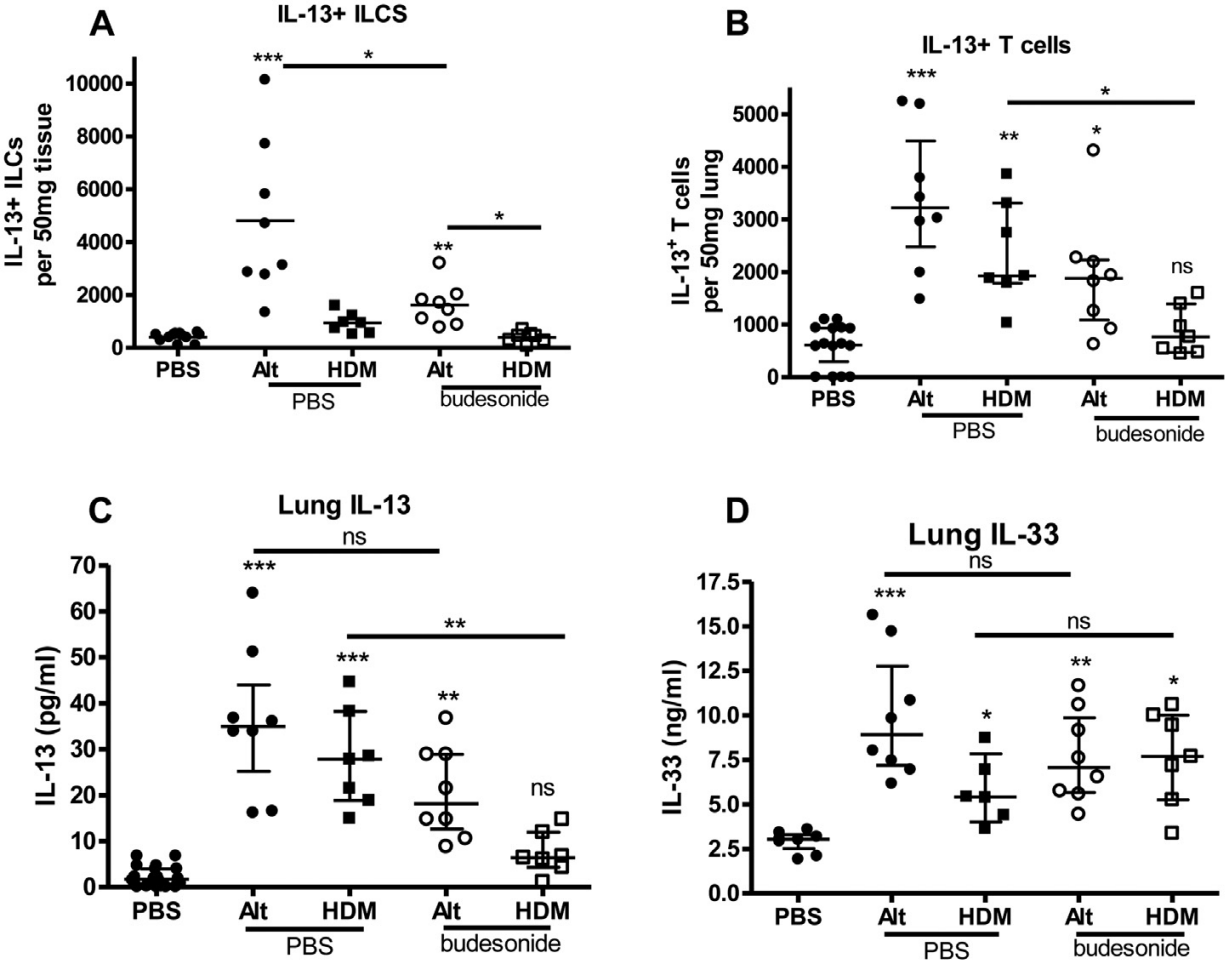
Steroids did not reduce IL-33 levels. Lung IL-13 levels and IL-13^+^ ILC and IL-13^+^ T-cell numbers only remained increased after steroids with *A alternata* exposure. Neonatal BALB/c mice were exposed to intranasal saline (PBS), HDM, or *A alternata (Alt)* for 3 weeks from day 3 of life, with either concomitant intranasal budesonide or saline (PBS). Lung IL-13^+^Lin^−^CD45^+^ICOS^+^ ILCs **(A)** and IL-13^+^CD3^+^CD4^+^ T cells **(B)** were quantified by means of flow cytometry. Levels of pulmonary IL-13 **(C)** and IL-33 **(D)** were measured by means of ELISA. There were 4 to 6 per group for the PBS group and 6 to 8 per group for the HDM or *A alternata* groups. *P < .05, **P < .01, and ***P < .001. *ns*, Not significant.

To determine why *A alternata* exposure resulted in steroid-resistant AHR, we quantified ILC2 and T_H_2 cell numbers in the lung after budesonide therapy. ILC2 numbers were not increased after HDM, but T_H_2 cell numbers were reduced to control levels after HDM exposure and steroids (Fig 6, *A* and *B*). Both ILC2 and T_H_2 cell numbers were increased after *A alternata* exposure and persisted despite steroids (Fig 6, *A* and *B*). Lung IL-13 levels also remained increased despite steroid therapy during *A alternata* exposure (Fig 6, *C*), correlating with persistent AHR (Fig 5, *B*). Importantly, lung IL-33 levels remained increased after steroid treatment after both *A alternata* and HDM exposure (Fig 6, *D*). Despite similarly increased IL-33 levels after steroid treatment after both *A alternata* and HDM exposure, a greater number of ILC2s and T_H_2 cells only persisted after *A alternata* exposure (Fig 6, *A* and *B*), suggesting *A alternata* induces stronger type 2 immunity than HDM, thus resulting in a more severe phenotype of allergic airways disease.

## Discussion

We have reported the clinical and bronchoscopic features of pediatric SAFS in a large cohort of patients and demonstrated a potential novel mechanism that might drive fungal sensitization in patients with this subphenotype of severe asthma. Children with SAFS had earlier asthma onset and more severe atopy and were prescribed more oral steroids than those not sensitized to fungi. They also had significantly more airway IL-33 and MMP-9 activity than those without fungal sensitization. However, there was no difference in symptoms, lung function, airway inflammation (either in tissue or BAL fluid), or airway remodeling, despite the higher levels of treatment in patients with SAFS. Using age-appropriate experimental models of chronic allergen exposure, we demonstrated increased IgE and IL-33 levels and ILC2 and T_H_2 cell numbers in response to *A alternata* exposure compared with those seen after HDM exposure. AHR and IL-13^+^ ILC2 and T_H_2 cell numbers remained increased despite use of steroids after fungal exposure, but these were significantly reduced in *A alternata*–exposed ST2^−/−^ mice, suggesting IL-33 is an important driver of SAFS. Therapeutic strategies to block IL-33 might have better clinical efficacy in patients with SAFS than the disappointing results of antifungal agents.

The principle strength of this study is the inclusion of a large number of children who have been carefully assessed after their basic asthma management was optimized.^17^ In contrast to adults,^24^ our children with SAFS were predominantly male. A recent report comprising children with asthma of a range of severities also showed those with fungal sensitization were more likely to have severe disease and higher IgE levels.^4^ IgE levels might not be strictly comparable between adults and children because the adult SAFS definition had an upper cutoff of 1000 IU/mL to ensure patients with ABPA were not included.^11^ ABPA is very rare in asthmatic children, and indeed, none of this group met the criteria for diagnosis; therefore we have not adopted this cutoff. None of our children were given a diagnosis of or treated for ABPA. We also extended these observations by quantifying atopy as the sum of sIgE or SPT results,^20^ again showing that children with SAFS were more atopic (to nonfungal inhalant allergens). We have only studied 3 of 7 fungi implicated in the adult definition of SAFS because we do not routinely test for sensitivity to other fungi. However, this could only have resulted in some children with SAFS being incorrectly placed in the control group, biasing against us finding any significant differences.

Although we did not see differences in inflammation in our children with and without SAFS, this is likely because the children with SAFS were prescribed more anti-inflammatory medications. Even though more children with SAFS were prescribed oral steroids, more than half still had BAL fluid and sputum eosinophilia (see Table E1), suggesting fungal sensitization might be associated with steroid resistance. We have previously shown that the innate cytokine IL-33 is relatively steroid resistant and that tissue expression is increased in children with severe asthma (regardless of fungal sensitization).^21^ In addition, IL-33 levels are significantly increased after a single dose of *A alternata* in an adult murine model.^16^ Therefore we determined BAL fluid and endobronchial biopsy specimen IL-33 levels in the children with severe asthma and showed that they were higher in patients with SAFS. This suggested that IL-33 might be important in mediating fungal sensitization in pediatric patients with severe asthma.

Because the children's data were cross-sectional and only very small airway samples could be obtained, studies to confirm the functional role of IL-33 in mediating SAFS could not be performed in the patients. Therefore we used an age-appropriate experimental model to assess mechanisms that might underlie fungal sensitization. Importantly, the children were polysensitized to numerous aeroallergens, but to determine specific mechanisms attributable only to fungal exposure, we compared a neonatal mouse model of chronic HDM exposure with chronic *A alternata* exposure. The dose of each allergen was equivalent because similar AHR was generated after the same duration of exposure. Even though the dose of HDM (20 μg) used was double our previously published dose^22^ and 4 times greater than the *A alternata* (5 μg) dose, significantly more inflammation and greater IgE and IL-33 levels were induced by *A alternata*. The neonatal model of fungal exposure mirrored the pediatric data, with increased IgE levels after *A alternata* compared with those after HDM exposure. There was also increased eosinophilia after *A alternata* exposure in the murine model. Although the effects of chronic *A alternata* challenge are associated with increased IL-33 levels^25^ in experimental models, direct comparisons of IL-33 release after fungal and nonfungal allergen exposure have not been made. Adult models of *A alternata* exposure have shown increased ILC numbers without increased T- or B-cell numbers.^26,27^ However, in our neonatal model *A alternata* increased pulmonary IL-33 levels concomitant with an increase in both T_H_2 cell and ILC2 numbers compared with HDM exposure. This suggests that both cell types might be induced by IL-33 in early life. A limitation was our inability to demonstrate an association between IL-33 levels and increased ILC2 or T_H_2 cell numbers in children with SAFS. However, the limited volume of BAL fluid obtained prevented detailed cellular characterization, and only mediators could be quantified.

**Fig E6:**
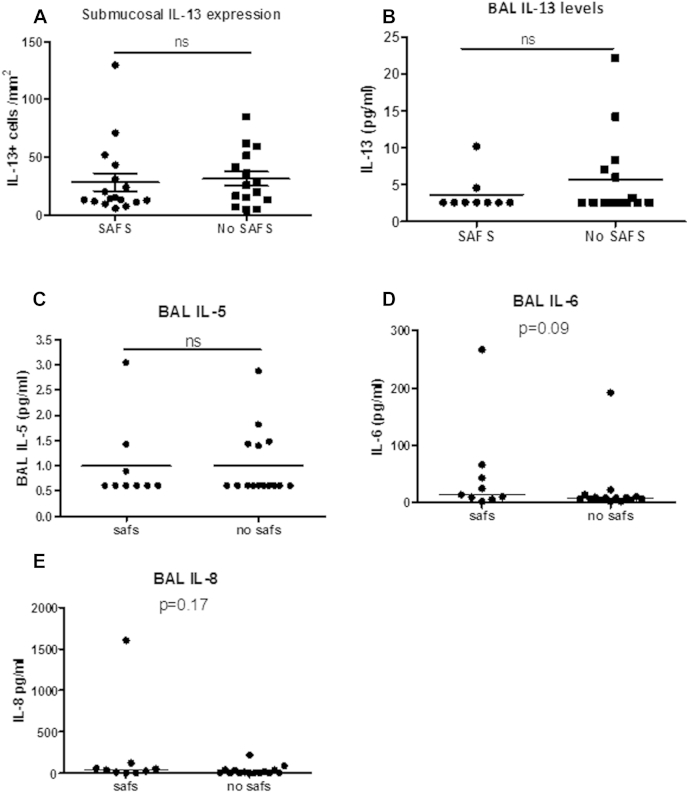
Endobronchial biopsy specimens from children aged 6 to 16 years with severe asthma were stained by means of immunohistochemistry for IL-13 expression. **A,** IL-13^+^ cells in the submucosa were counted and expressed per area of submucosa in children with SAFS compared with children with severe asthma without fungal sensitization *(no SAFS)*. **B-E,** IL-13 (Fig E6, *B*), IL-5 (Fig E6, *C*), IL-6 (Fig E6, *D*), and IL-8 (Fig E6, *E*) levels in BAL fluid from children with SAFS and non-SAFS were quantified by using the Luminex multiplex assay (Bio-Rad). *ns*, Not significant.

For the first time, we have shown that chronic *A alternata* exposure results in relatively steroid-resistant AHR in neonatal mice. Treatment with daily inhaled steroids in a prevention regimen at a dose equivalent to that used in children with STRA (before allergic airways disease was established) did not alter AHR in the *A alternata* model. In contrast, AHR was completely abrogated with the use of steroids in a similar regimen with HDM exposure. We have previously shown that steroids in a therapeutic regimen only partially reduce AHR in our neonatal HDM model,^21^ but with the current prevention regimen, HDM-induced AHR is steroid sensitive, whereas *A alternata*–induced AHR is steroid resistant. Despite steroids, pulmonary IL-33 levels and IL-13^+^ T_H_2 cell and ILC2 numbers remained increased after *A alternata* exposure. Although IL-33 levels remained similarly increased after steroids during exposure to both allergens, significantly more ILC2s were induced by *A alternata* than HDM, and although there was a partial reduction in ILC2 numbers after steroids, they remained significantly higher than in HDM-exposed mice after steroids, thus resulting in higher lung IL-13 levels and AHR. Interestingly, BAL fluid IL-13 was completely abrogated by steroids after *A alternata* exposure (data not shown), but we know that lung IL-13 levels most closely reflect AHR in our neonatal model.^22^ This finding in BAL fluid concurs with our previous clinical findings of very low/undetectable BAL fluid IL-13 levels in children with STRA.^1^ We have also assessed levels of IL-13 and other T_H_2 cytokines in BAL fluid from the children according to fungal sensitization status, but levels did not correlate with SAFS (see Fig E6 in this article's Online Repository at www.jacionline.org).

Although previous experimental studies reported an association between IL-33 levels and fungal exposure,^16,25,26,28,29^ to our knowledge, this is the first to show directly, using ST2^−/−^ mice, that chronic fungal sensitization is mediated by IL-33. In addition, a relationship between steroid resistance and fungal sensitization has not been reported. We have also confirmed such an association in patients with STRA, thus identifying a novel mechanism underlying SAFS and a potential novel therapeutic target. IL-33 is likely a specific therapeutic target for pediatric SAFS because we have been unable to detect increased levels of the other innate cytokines IL-25 or thymic stromal lymphopoietin in either our neonatal mouse model of allergen exposure or in BAL fluid from children with STRA.^21^ This is important because the only SAFS-specific therapeutic approach adopted to date has been the use of antifungal agents. However, there is a risk of adverse interactions between itraconazole and inhaled corticosteroids that might lead to iatrogenic Cushing syndrome.^30^ Moreover, antifungal therapy in patients with SAFS shows variable efficacy whereby itraconazole resulted in improved quality of life^14^ but was also associated with a 40% failure rate.^13^ Although voriconazole and posaconazole as second- and third-line antifungal agents might be promising therapies,^13^ a randomized placebo-controlled trial of voriconazole in adults with asthma sensitized to *A fumigatus* with at least 2 severe asthma exacerbations in the previous year showed no benefit on exacerbations or quality of life.^15^ There are no interventional studies of antifungal agents in children with SAFS. However, given the varying effect from adult studies to date and that only one of the 82 children included in this study had positive fungal growth from BAL fluid, antifungal therapy might not be beneficial. We did not introduce antifungal agents into our neonatal mouse model because oral itraconazole during HDM exposure of adult mice plus short-term *A fumigatus* inhalation reduced eosinophilic and neutrophilic inflammation but had no effect on IL-5 or IL-13,^31^ and our previous data show IL-13 is most closely related to AHR.^22^ Interestingly, additional treatment with the steroid dexamethasone also did not reduce IL-13 levels after HDM and *A fumigatus* exposure, suggesting agreement with our data that any fungal exposure causes reduced steroid sensitivity.

An important feature of SAFS apparent from both our human and murine data was significantly increased serum IgE levels. Therefore anti-IgE mAb therapy (omalizumab) could be proposed as a preferred treatment option in patients with SAFS. However, in our cohort there was no significant difference in clinical response to omalizumab between patients with and without fungal sensitization in the relatively small numbers treated (Table I). This suggests the effects of blocking IgE are not specific enough to affect the disease severity and AHR caused by fungal sensitization.

We have previously shown that children with STRA have increased airway remodeling compared with that seen in nonasthmatic control subjects.^1^ However, when we looked at reticular basement membrane thickness and smooth muscle mass, there were no differences between children with and without SAFS. This might be because all children had severe disease and established remodeling very early, which is similar to the situation in adult patients.^32^ However, in a recent adult fungal exacerbation murine model, a relationship between IL-33 levels and levels of the protease and proremodeling mediator MMP-9 was demonstrated.^16^ Therefore MMP-9 activity was measured in pediatric BAL fluid and showed significantly increased levels in children with SAFS. This was confirmed in BAL fluid from *A alternata*–exposed neonatal mice. MMP-9 is a downstream target of IL-33,^16^ and activity is increased in sputum from patients with severe asthma who are unresponsive to steroid therapy.^33^ Both IL-33 and MMP-9 activity was increased in the BAL fluid of our patients. Because we have previously shown an association among IL-33 levels, airway remodeling, and steroid resistance, higher IL-33 levels in patients with SAFS might contribute to airway remodeling through MMP-9. MMP-9 is a sensitive indicator and mediator of airway remodeling activity^34^ rather than established remodeling and can be more easily measured in sputum and BAL samples^33,35^ than IL-33, suggesting it could be used as a soluble biomarker to assess therapeutic response to blocking IL-33 in patients with SAFS.

It is acknowledged that the mechanisms underlying SAFS are complex and the role of immunomodulating agents requires investigation.^36^ However, we report a novel association between steroid-resistant IL-33–induced AHR mediated by ILC2 and T_H_2 cells and the development of SAFS and propose IL-33 as a potential therapeutic target for this subphenotype of severe asthma.

**Key messages**

• Pediatric SAFS is associated with increased IL-33 levels and increased oral steroid therapy.
• Neonatal allergic airways disease induced by chronic *A alternata* exposure is steroid resistant, with higher IL-33–mediated ILC2 and T_H_2 cell numbers compared with those after HDM exposure.
• The steroid-resistant cytokine IL-33 might be a novel therapeutic target for SAFS.

## Figures and Tables

**Table E1:** Inflammation and remodeling in children with STRA with and without fungal sensitization

Parameter	STRA with fungal sensitization (n = 38)	Non–fungus-sensitized STRA (n = 44)	*P* value
Feno (ppb), median (range)	30.5 (4.8-170), n = 38	55.7 (3.4-231), n = 40	.012
Sputum eosinophils (%), median (range)	4 (0-67), n = 20	6.9 (0-92), n = 22	.3
Sputum neutrophils (%), median (range)	36 (5-86), n = 20	24 (0-97), n = 22	.5
BAL eosinophils (%), median (range)	2.7 (0-16), n = 31	2.3 (0-51), n = 33	NS
BAL eosinophilia (>1.19%), no./total no. with data available (%)	22/36 (55)	29/40 (72.5)	NS
BAL neutrophils (%), median (range)	4 (0-75), n = 31	3.3 (0-74), n = 33	NS
BAL neutrophilia (>3.5%), no./total no. with data available (%)	19/36 (52.8)	18/38 (47.4)	NS
RBM thickness (μm), median (range)	7.12 (4.8-13.1), n = 24	7.7 (4.3-11.4), n = 33	NS
ASM mass Vv (sm/subepithelium), median (range)	0.22 (0.05-0.65), n = 19	0.16 (0-0.55), n = 28	NS
ASM mass Vs (sm/subepithelium), median (range)	26.3 (6.8-83.5), n = 14	18.9 (0.7-81.6), n = 20	NS
Epithelial shedding (% epithelial loss), median (range)	90.3 (68.9-100), n = 23	92.5 (17.7-100), n = 29	NS

*ASM*, Airway smooth muscle; *Feno*, fraction of exhaled nitric oxide; *NS*, not significant; *RBM*, reticular basement membrane; *Vs*, subepithelial volume fraction of airway smooth muscle indexed to surface area of RBM; *Vv*, subepithelial volume fraction of airway smooth muscle indexed to subepithelial tissue.

**Table I:** Patients' demographics

Characteristics	STRA with SAFS (n = 38)	Non–fungus-sensitized STRA (n = 44)	*P* value
Sex (male/female)	30/8 (male = 78.9%)	22/22 (male = 50%)	.007
Age at symptom onset (y), median (range)	0.42 (0-12.5), n = 36)	1 (0-12.5), n = 43	.015
Atopy, no./total no. with data available (%)	37/38 (97.4)	33/44 (75)	.004
Total IgE (IU/mL), median (range)	634 (24-6737), n = 37	298 (7-4610), n = 43	.015
Sum of nonfungal inhalant SPT wheal diameter (mm), median (range)	16 (3-38), n = 27	9 (0-29), n = 41	.008
Sum of nonfungal inhalant sIgE (IU/mL), median (range)	68.4 (0-287), n = 33	30.8 (0-220.5)	.02
Body mass index (kg/m^2^), median (range)	19.7 (7.1-29.7), n = 18	18.3 (14.9-36.6), n = 28	NS
Successful trial of omalizumab, no./total no. with data available (%)	8/10 (80)	11/18 (61)	NS
Prescribed maintenance OCS, no./total no. with data available (%)	16/38 (42.1)	6/42 (14.3)	.02
ICS dose (budesonide equivalent μg/d), median (range)	1500 (800-3000), n = 38	1600 (800-4800), n = 44	NS
ACT score, median (range)	13 (6-23), n = 34	13 (6-25), n = 40	NS
FEV_1_ (% predicted, median (range)	71 (29-121), n = 38	71.5 (34-99), n = 42	NS
FVC (% predicted), median (range)	94.5 (36-133), n = 38	91.3 (57-123), n = 42	NS

*ACT*, Asthma Control Test; *FVC*, forced vital capacity; *ICS*, inhaled corticosteroids; *NS*, not significant; *OCS*, oral corticosteroids.
